# Labor-Associated Gene Expression in the Human Uterine Fundus, Lower Segment, and Cervix

**DOI:** 10.1371/journal.pmed.0030169

**Published:** 2006-06-13

**Authors:** Radek Bukowski, Gary D. V Hankins, George R Saade, Garland D Anderson, Steven Thornton

**Affiliations:** **1**Department of Obstetrics and Gynecology, University of Texas Medical Branch at Galveston, Galveston, Texas; **2**Warwick Medical School, University of Warwick, Coventry, United Kingdom; Imperial College LondonUnited Kingdom

## Abstract

**Background:**

Preterm labor, failure to progress, and postpartum hemorrhage are the common causes of maternal and neonatal mortality or morbidity. All result from defects in the complex mechanisms controlling labor, which coordinate changes in the uterine fundus, lower segment, and cervix. We aimed to assess labor-associated gene expression profiles in these functionally distinct areas of the human uterus by using microarrays.

**Methods and Findings:**

Samples of uterine fundus, lower segment, and cervix were obtained from patients at term (mean ± SD = 39.1 ± 0.5 wk) prior to the onset of labor ( *n =* 6), or in active phase of labor with spontaneous onset ( *n =* 7). Expression of 12,626 genes was evaluated using microarrays (Human Genome U95A; Affymetrix) and compared between labor and non-labor samples. Genes with the largest labor-associated change and the lowest variability in expression are likely to be fundamental for parturition, so gene expression was ranked accordingly. From 500 genes with the highest rank we identified genes with similar expression profiles using two independent clustering techniques. Sets of genes with a probability of chance grouping by both techniques less than 0.01 represented 71.2%, 81.8%, and 79.8% of the 500 genes in the fundus, lower segment, and cervix, respectively. We identified 14, 14, and 12 those sets of genes in the fundus, lower segment, and cervix, respectively. This enabled networks of co-regulated and co-expressed genes to be discovered. Many genes within the same cluster shared similar functions or had functions pertinent to the process of labor.

**Conclusions:**

Our results provide support for many of the established processes of parturition and also describe novel-to-labor genes not previously associated with this process. The elucidation of these mechanisms likely to be fundamental for controlling labor is an important prerequisite to the development of effective treatments for major obstetric problems—including prematurity, with its long-term consequences to the health of mother and offspring.

## Editors' Summary

**Background.:**

Childbirth, or labor, although a basic event in life, is actually a complex process that involves three parts of the uterus (womb) working together to expel the baby. One particularly important part of the process, which is poorly understood, is how labor begins. The actual changes that occur in the uterus once labor has begun are well known, and include contractions in the muscle of the uterus wall (the myometrium) and dilation of the cervix (the neck of the womb). Some of the triggers for these changes are also known: for example, in non-primate animals changes in the blood levels of the hormones estrogen and progesterone and changes in the membranes that surround the fetus. Previous studies have suggested that these effects are likely, in turn, to be triggered by changes in many genes, but exactly which ones is not clear.

**Why Was This Study Done?:**

Learning more about which genes are important in the various stages of labor may help to design treatments for the various problems that occur in labor (such as failure of labor to begin, or, alternatively, preterm labor). Little is known about the genes that trigger, or are necessary for, labor to start and to continue in a coordinated fashion. A technology known as DNA microarrays allows researchers to take a sample from any part of the body and use it to look at how active many thousands of genes are, all at the same time. By analyzing these results, it is possible to suggest either single genes or groups of genes that may be important in a particular process.

**What Did the Researchers Do and Find?:**

The authors took samples from the uterus top, lower part, and cervix of six women before their labor started, and seven from those whose labor had started. All women were having cesarean sections either for medically indicated reasons, or for choice. Then, in each of the samples in each woman, they looked at 12,626 known genes to see how active they were (scientists call these active genes “expressed”). They found that the changes in gene expression were not, generally, the same across the three parts of the uterus. Of the 500 genes with the largest change in expression, 28 were common to both the upper and lower parts of the uterus, and this small group of genes may be important in labor in both the upper and lower parts of the uterus. The authors also classified the 500 genes into related groups, and they believe that these relationships may be important in controlling how labor happens.

**What Do These Findings Mean?:**

Identifying new genes or groups of genes involved in labor is important for understanding how labor occurs. One limitation of this study is the small number of women who were studied—which is understandable, given the difficulty of obtaining such samples—and the differences between the women studied. Another difficulty with such studies is that the methods used to analyze the expression patterns can affect the results. However, as is the custom with these types of studies, all the results were placed in a public database so anyone can look at them and, if they wish, do further analyses. In a related Perspective article that was commissioned to comment on this paper, Roberto Romero, one of the original reviewers of the paper, has done just that. He finds that there were differences in the results of his analyses and those of the authors'. He goes on to discuss the question of how hard it is to use these techniques to look at complex problems, such as how labor starts. Clearly, much more work needs to be done before it is clear what all these results really mean. Nonetheless, these studies have the potential to help to understand more about the basic science behind labor.

**Additional Information.:**

**Please access these Web sites via the online version of this summary at** http://dx.doi.org/10.1371/journal.pmed.0030169.

• Medline Plus has a page of links on childbirth

## Abstract 3

Radek Bukowski and colleagues used microarrays to assess labor-associated gene expression profiles in the uterus and discover networks of co-regulated and co-expressed genes.

## Introduction

The onset and progression of normal labor involves complex maternal and fetal interactions leading to dilation of the cervix and coordinated uterine contractions.

Temporal disruption of this process can lead to preterm delivery, and ineffective uterine contractility can cause failure to progress in labor or postpartum hemorrhage. These problems have important consequences. Preterm delivery is a major cause of neonatal mortality and morbidity, including long-term neurological impairment [ 1]. Failure to progress in labor may lead to maternal morbidity and/or caesarean section [ 2] with its inherent risks, and postpartum hemorrhage is one of the main causes of maternal mortality worldwide [ 3].

Pregnancy is maintained by myometrial quiescence and cervical resistance. Toward term, there is a progressive activation of the myometrium and the cervix ripens in preparation for labor. Labor is associated with dramatic changes in myometrial contractions and cervical dilation resulting from increased stimulatory and reduced inhibitory processes [ 4]. These effects are due to simultaneous and interdependent changes in cellular proteins initiated by a multitude of genes. The molecular processes are spatially coordinated to result in uterine contractions with simultaneous cervical dilation. Additional spatial organization of contractile processes within the myometrium results in increased contractility of the fundus compared to the lower segment [ 4– 7].

The specific changes in gene expression that cause these temporal and spatial effects are largely unknown. Our hypothesis is that labor results from the simultaneous change in expression of a large number of genes that are organized into co-regulated networks. We examined the labor-associated gene expression changes in the human fundus, lower segment, and cervix using Affymetrix genome DNA microarrays.

## Methods

### Sample Collection

Tissue was obtained from patients undergoing cesarean section and sterilization without medical or obstetrical complications of pregnancy and who were not exposed to medications immediately before enrollment. The procedure was approved by the Institutional Review Board and Coventry Research Ethics Committee (IRB 00-022, CREC 062/05/01), and informed consent was obtained from all eligible patients. Samples were obtained from patients at term (mean ± SD = 39.1 ± 0.5 wk) prior to the onset of labor ( *n =* 6), or in active phase of labor with spontaneous onset ( *n =* 7). Labor was defined as cervical dilatation of >3 cm or progressive dilation accompanied by regular uterine contractions. Patients not in labor were delivered by cesarean section on maternal request following counseling by obstetrician because of previous cesarean section or because an abnormal fetal presentation in the index pregnancy made vaginal delivery unsafe. Patients in labor had failure to progress despite adequate contractility or fetal intolerance of labor.

For each patient, samples (approximately 1 cm ^3^) were taken from the uterine fundus (the outside surface of the uterus that does not include decidua), the lower segment at the upper edge of the incision, and the anterior lip of the cervix, through the vagina. We have previously shown that our lower-segment samples are more than 98% myometrial smooth muscle [ 8]. In one patient, a biopsy of the cervix could not be obtained. Samples were immediately snap-frozen in liquid nitrogen and stored at −80 °C.

### Microarray Analysis

All samples were analyzed separately without pooling of extracted RNA. RNA isolation was performed using TRIzol Reagent (Gibco BRL Life Technologies, San Diego, California, United States) followed by phenol extraction and ethanol precipitation. Genomic contamination was removed by on-column treatment of RNA samples with DNAse (27 Kunitz units) for 20 min at 20 °C (Qiagen, Valencia, California, United States).

Isolated total RNA was quantified by spectrophotometry. Double-stranded cDNA was synthesized from total RNA using T7-(dT) _24_ oligomer primer (Genset Corp., La Jolla, California, United States) and Superscript II Reverse Transcriptase (Gibco BRL Life Technologies). For complete recovery of the cDNA, samples were subjected to phase-lock gel phenol-chloroform extraction and ethanol precipitation. 1 μg of cDNA was used for an in vitro transcription reaction, which involved the synthesis of the biotin-labeled cRNA from the cDNA with biotinylated CTP and UTP (Enzo Life Sciences, Farmingdale, New York, United States). The biotin-labeled RNA fragments were then hybridized to microarray chips (Human Genome U95A; Affymetrix, Santa Clara, California, United States). Microarrays from several different lots were used to analyze samples. Different lots of microarrays will increase variability of findings but will minimize the chance of a bias or systematic error associated with a certain lot and resulting in false positive and negative findings. The chips were washed, stained on a fluidic station, and scanned by confocal microscope. Each chip was used only once. The average difference intensity was calculated and describes the difference between the intensities of emitted light from hybridized matched probes and their mismatched controls. The average for the 20 probes and their controls are calculated for each gene.

To allow comparison between genes and patients, average difference intensities were converted into percentiles and *z*-scores. To allow comparison between genes, the differences in RNA hybridizations between probes and controls were normalized by conversion into percentiles. To allow comparison of samples (chip to chip), the percentile values were converted into *z*-scores for a given gene expression across all samples (expression value – mean/standard deviation).

Identification of the putative gene functions used NetAffx (Affymetrix), an integrated online resource from the GenBank, UniGene, and Gene Ontology databases, and the Ingenuity database ( http://www.ingenuity.com).

### Statistical Methods

All samples were analyzed separately. To identify genes demonstrating a maximal labor-associated change in expression, the *p-*value was calculated by Student's *t*-test. This *p*-value was used as a measure of the magnitude of the change and inter-subject variability rather than to determine significance. Genes were ordered according to the *p-*value. The 500 genes in each of the fundus, lower segment, and cervix with the lowest *p-*values were selected for further analysis.

These genes were clustered using two different techniques: K-means and hierarchical. K-means is a non-hierarchical clustering method that groups data points into a predetermined number of clusters. It is an iterative process in which each gene profile is assigned to the closest centroid, which is the center point of a cluster. The centroid is then recomputed until a steady state has been reached. Euclidian distance was used as a similarity measure for gene profiles. Centroids were initialized using a data-based centroid search. The number of clusters was selected to provide a wide range of genes per cluster without uninformative clusters containing no or single genes.

**Figure 1:**
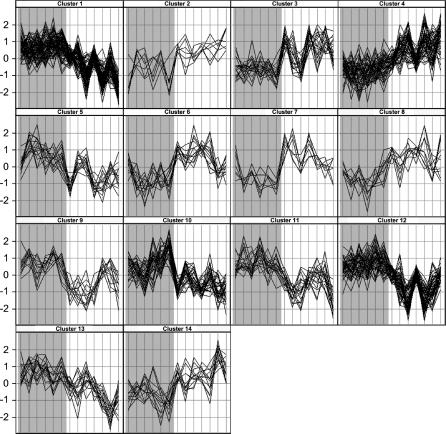
Profiles of Gene Expression in the Uterine Fundus from Women before or after the Onset of Labor Each panel shows profiles of the genes within one of the clusters determined jointly by K-means and hierarchical clustering. On the *x*-axis, samples from individual patients are arranged and represented by vertical lines. Non-labor samples (gray background) are shown on the left and labor (white background) on the right. The *y*-axis represents the level of gene expression as a number of standard deviations from the mean of all observations for each gene ( *z*-score).

**Figure 2:**
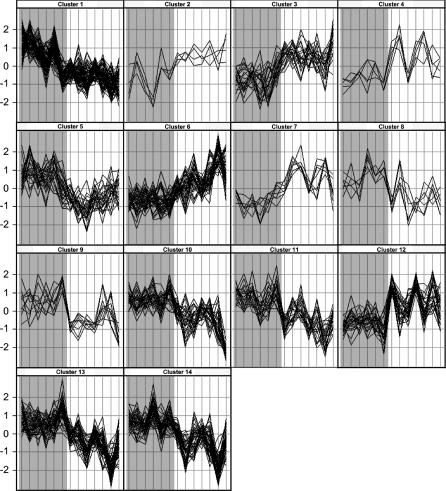
Profiles of Gene Expression in the Uterine Lower Segment from Women before or after the Onset of Labor Each panel shows profiles of the genes within one of the clusters determined jointly by K-means and hierarchical clustering. On the *x*-axis, samples from individual patients are arranged and represented by vertical lines. Non-labor samples (gray background) are shown on the left and labor (white background) on the right. The *y*-axis represents the level of gene expression as a number of standard deviations from the mean of all observations for each gene ( *z*-score).

**Figure 3:**
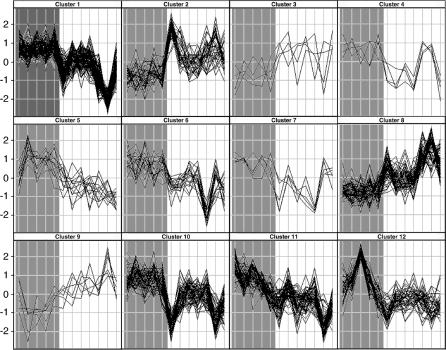
Profiles of Gene Expression in the Uterine Cervix from Women before or after the Onset of Labor Each panel shows profiles of the genes within one of the clusters determined jointly by K-means and hierarchical clustering. On the *x*-axis, samples from individual patients are arranged and represented by vertical lines. Non-labor samples (gray background) are shown on the left and labor (white background) on the right. The *y*-axis represents the level of gene expression as a number of standard deviations from the mean of all observations for each gene ( *z*-score).

Hierarchical clustering arranges the genes on a treelike system. Clusters are merged if the expression profiles are similar. The similarity between gene expression profiles was calculated using Euclidian distance and between clusters using unweighted pair-group method with arithmetic mean. Genes clustered together by both techniques were identified. Coincidence testing [ 9] was used to determine whether co-clustering was likely to have arisen by chance. Figures 1– 3 depict clusters of genes grouped together by both methods where probability of chance co-clustering was *p* < 0.01.

Within each of the coincidence clusters, we identified genes with functions similar to other genes within the same cluster or functions pertinent to the process of labor. For this purpose we used an interactive database of gene functions and interactions (Ingenuity pathway analysis) and biological knowledge database ( http://www.ingenuity.com).

### Validation of the Microarray Findings

Labor-associated changes in the expression of selected genes were examined using RT-PCR. Reaction products were separated, detected, and quantified with chip-based gel electrophoresis (Agilent 2100 bioanalyzer; Agilent Technologies, Palo Alto, California, United States) as described previously [ 10]. The number of PCR cycles (35) was selected from the linear portions of the dynamic ranges of amplification. The quantification and sizing coefficients of variation are <6.7% and <2.1 %, respectively [ 11]. All mRNA abundance data were expressed relative to constitutively expressed 18S rRNA. The non-labor and labor samples were compared using the Mann-Whitney U test.

## Results

We analyzed the expression of 12,626 known genes in biopsies taken from the fundus, lower segment, and cervix either before ( *n =* 6) or after the onset of labor ( *n =* 7). The expression of each gene was quantified using an Affymetrix gene microarray. Student's *t-*test was used to determine the *p-*value for the difference in gene expression in samples taken before or after labor. This test identifies those genes with the largest labor-associated change in expression and the lowest variability. The 500 genes with the lowest *p-*values were selected from fundus ( Dataset S1), lower segment ( Dataset S2), and cervix ( Dataset S3). Of the 500 genes with the largest change in expression, 28 were common to both the fundus and lower segment. This finding suggests that a small core of genes is associated with labor in both the upper and lower segments of the uterus. Most changes in gene expression however, are not common, supporting the hypothesis of differential spatial regulation [ 12]. In both areas of the uterus, labor was associated with an overall reduction, rather than increase in gene expression. Expression was reduced in 71.4%, 72.4%, and 79.2% of the 500 genes after the onset of labor in the fundus, lower segment, and cervix, respectively.

Since many genes in reproductive tissues may be co-regulated or interdependent, we identified groups of genes with similar expression profiles. We placed the selected 500 genes into one of ten clusters. Two different techniques were used: K-means and hierarchical. The number of genes per cluster determined by K-means ranged between 31–83, 26–93, and 115–102 for fundus, lower segment, and cervix, respectively. The corresponding number of genes for each cluster by hierarchical clustering was 3–239, 3–181, and 2–333, respectively. To further refine the gene groups we determined those genes which were co-clustered using both techniques. Coincidence testing was done to determine the probability that each set of genes was co-clustered using both techniques by chance. Sets of genes with a probability of chance grouping less than 0.01 were analyzed further. These sets represented 71.2%, 81.8%, and 79.8% of the 500 genes in the fundus, lower segment, and cervix, respectively. Since genes grouped by one technique can also be grouped in any of the ten groups from the second technique, there are 100 possible co-clusters. We found only 14, 14, and 12 clusters in the fundus, lower segment, and cervix, respectively, suggesting that these co-clusters are likely to represent interdependent or co-regulated genes. Examples of genes clustered together by both techniques are shown in Tables 1– 3. (Complete data are available online and can be accessed at http://www.ebi.ac.uk/arrayexpress, accession number E-MEXP-106).

**Table 1:**
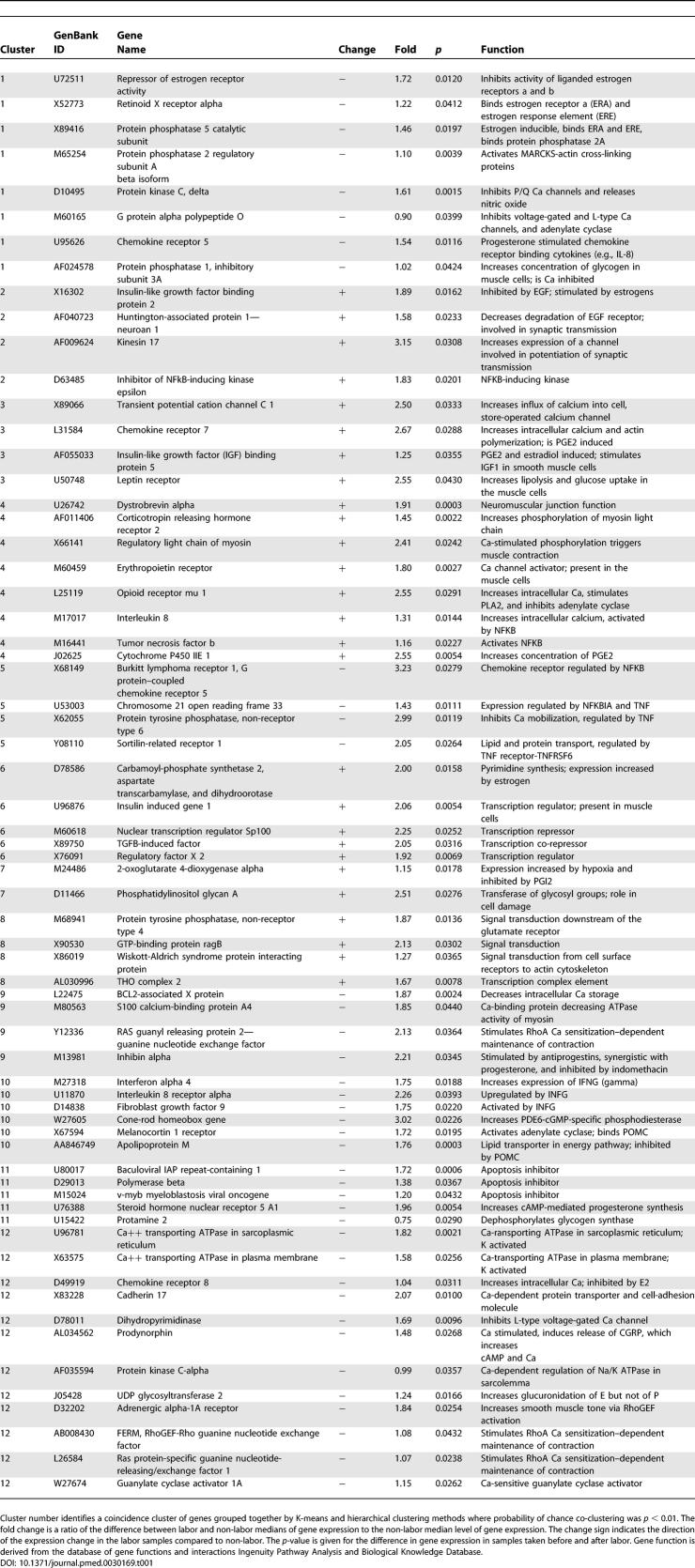
Fundus: Selected Genes within Each of the Coincidence Clusters with Functions Similar to Other Genes within the Same Cluster or Functions Pertinent to the Process of Labor

Examination of the data raises some interesting hypotheses. For example, in the lower segment, expression of the genes for the nuclear binding protein C/EBP, TNF receptor, alpha 1A–adrenergic receptor, phospholipase A2 (group IIA), and G protein–coupled receptor 18 have similar expression profiles. In the fundus, the expression of repressor of estrogen receptor activity (REA) is reduced, while prothymosin alpha remains unchanged with labor. The two genes constitute one of the reported regulatory pathways of estrogen receptor alpha (ERA) activity [ 13].

Numerous genes have been reported to change in expression dramatically in reproductive tissues at the onset of labor. Our results are consistent with these previous results and demonstrate in the lower segment a marked increase in expression of the genes for beta-adrenergic receptor kinase 2 [ 14], phospholipase A2 IIA [ 15], and calcium ion–transporting ATPase 2 [ 16]. Furthermore, there was a reduction in expression of regulator of G protein signaling 2 [ 17], calcitonin receptor activity–modifying protein 2 [ 18], and protein kinase C [ 19]. Nevertheless, some genes that would be expected to demonstrate a marked labor-associated increase, such as prostaglandin receptor EP 4 [ 20], were not selected by our technique, possibly due to a large inter-patient variability in expression. Since other genes' expression patterns were consistent with prior findings, this variability may reflect gene polymorphism.

**Figure 4:**
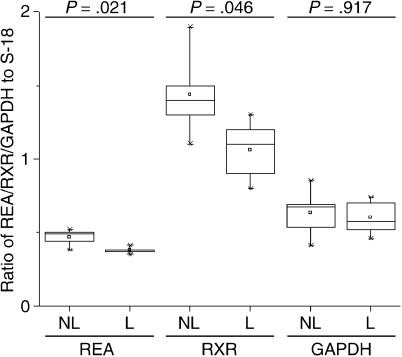
Validation of the Microarray Findings by RT-PCR Relative abundance of mRNA, normalized to S-18, encoding the gene for REA, RXR, and GAPDH in the myometrium obtained from the uterine fundus in five non-laboring (NL) and five spontaneously laboring (L) patients. Box limits represent 25th and 75th quartiles, line within the box represents median, and whiskers represent 5th and 95th percentiles.

Expression changes of REA, retinoid X receptor alpha (RXR), and glyceraldehyde-3-phosphate dehydrogenase (GAPDH) in association with labor in the uterine fundus corresponded to the findings of microarray experiments. Both REA and RXR expressions decreased in labor, while expression of GAPDH remained unchanged ( Figure 4).

## Discussion

Our results demonstrate labor-associated changes in gene expression in three functionally important areas of the human uterus. The primary objective of this study was to identify novel-to-labor genes important for the process of parturition. The second main objective was to identify groups of genes with similar expression profiles in order to recognize those with common regulatory mechanisms. Rather than providing a list of genes, this results provides a map of gene interactions in labor. We postulated that the onset of labor is likely to be caused by a reduction in inhibitory and an increase in stimulatory processes, and our data support this theory; for example, we demonstrated that in the lower segment in labor expression of genes for the stimulatory tumor necrosis factor receptor is increased, whereas that of the relaxatory potassium channel is reduced.

The study was specifically designed to investigate gene expression in human labor because the mechanisms of labor vary between species. Previous gene array studies have documented changes in expression in a rodent model [ 21, 22]. Such animal models are useful since variability is reduced because of the animals' similar genotypes and exposure to a controlled environment. Expression data from such studies can be compared and contrasted with those from human tissue, thus providing an insight into the similarities and differences between species. However, we consider that data from human studies are the most important for understanding human physiology.

Previous human gene array data [ 22, 23], has marked differences in methodology from our study. Bethin and colleagues [ 22] determined the expression profile in human extracts obtained either preterm, prior to labor or preterm, and at term following the onset of labor. In contrast, we designed our study to specifically determine labor-associated alterations and to exclude the marked changes in expression at the end of pregnancy. A further difference in our study was that we analyzed human uterine samples from all three functionally distinct areas of the uterus in the same women. It is the cooperation of these components of the uterus (contraction of the fundus, relaxation of the lower segment, and dilation of the cervix) that result in the process of labor. Our study also differed in that the method of analysis and sample size enabled the individual variation between women to be taken into account—i.e., to preserve these characteristics samples were not pooled. This individual analysis enabled the expression of each gene to be identified in each sample. The genes were then grouped into clusters based on their similarity of expression across individual samples. This similarity of genes' expressions in different samples dramatically increases the power of the cluster analysis and is possible only because the individual sample characteristics are maintained. However, one limitation of an individual analysis is that individual variation in expression in human tissue is likely to be high, not only because there are marked genetic and environmental effects but also because the time to the onset of spontaneous labor in non-labor samples is not known.

Aguan and colleagues utilized a different experimental design and methodology to investigate gene expression in the lower uterine segment before and after the onset of labor [ 23]. The type of array, number of investigated genes (588), and normalization procedures make valid comparison with our study difficult. The studies differ also in how the fold change in the gene expression calculation was done. However, there are several consistent changes in gene expression. For example, we demonstrated a 91% decrease in G protein–coupled receptor 161 in lower-segment samples, which is consistent with the 84% reduction reported by Aguan et al. We also demonstrated consistent changes in guanine nucleotide binding protein alpha expression.

Chan and colleagues [ 24] studied uterine samples in labor using a subtractive hybridization technique. Although this study used a different technique from ours, and the number of genes upregulated in labor was small, their findings have shown a consistent with our results, significant increase in the expression of interleukin-8.

Gene array data provide a wealth of information, which presents unique analytical challenges. We determined expression in six samples taken before and seven after the onset of labor at term. In order to compare the differences in samples taken before and after labor, the *t*-test for the difference in expression was performed and the *p-*value was calculated. The genes were ranked according to this value, not to determine significance, which would be inappropriate for this number of comparisons, but to determine genes that demonstrated the greatest and most consistent change in labor. We did not correct for multiple comparisons since the expression of the different genes is not independently regulated. This method of analysis is likely to provide more consistent data than techniques using fewer samples, duplicate arrays on the same samples, or identification of an arbitrary change in expression [ 21, 22]. By this method, any difference in expression during labor of those genes with the smallest *p-*values is unlikely to have arisen by chance due to observer and instrument variability. Hence, the genes with the most consistent change in expression during labor are most likely to have an important function, although current methodology does not allow primary changes in expression to be distinguished from those secondary to increased contractility.

Although we cannot exclude false positives and negatives, the lower the *p-*value the smaller the probability of a false positive results. However, as the number of genes selected increases, so does the chance of inclusion of a false positive result, while the chance of a false negative one decreases. False negative results may also occur due to wide inter-patient variability. There are many potential causes of inter-patient variability. Particularly important is that it cannot be determined in non-laboring patients when parturition would otherwise commence—that is, how close to the onset of labor a non-laboring patient is.

It is likely that many changes in gene expression precede the clinical signs of labor: for example, the steroid hormones estrogen and progesterone are fundamentally important for the maintenance of pregnancy and the onset of labor [ 4, 7]. In some species, such as the sheep, pregnancy is maintained by progesterone and labor is caused by a dramatic fall in progesterone. The decrease in progesterone concentration increases the estrogen/progesterone ratio leading to contractions [ 25]. A fall in plasma progesterone has not been demonstrated in women, although administration of antiprogestins can induce labor [ 26]. This suggests that the mechanism may be slightly different in women.

Our demonstration of a fall in the expression of a modulator of estrogen receptor activity provides a mechanism whereby the functional estrogen/progesterone ratio could be increased without a change in plasma concentration of either. REA is a protein that competitively and selectively binds to the nuclear receptor reducing its function [ 13]. Although identified in breast cancer and placental cells, this modulator has not been described in the human myometrium. REA and RXR (which also inhibits estrogen activity) were both clustered into one group based on their decreased expression pattern in labor. The expression of prothymosin alpha (an antagonist of REA) is unchanged in labor and further supports this hypothesis. Jointly, they demonstrate existence of a pathway that may represent a novel mechanism of uterine control [ 13].

During labor there are concomitant physiological changes in the fundus, lower uterine segment, and cervix. The fundus generates coordinated forceful uterine contractions while the contractile lower segment elongates over the presenting part. The cervix undergoes softening in late pregnancy with a dramatic shortening and dilation during labor. Our data demonstrate related marked spatial differences in gene expression, consistent with previous publications using alternative techniques for quantification [ 12]. Some of these differences in gene expression may, however, be due to cell type. We have previously demonstrated that more than 98% of cells in our lower segment biopsies are myometrial [ 8], and fundal samples were taken from the peritoneal (outer) surface to prevent decidual contamination. It is therefore unlikely that changes in gene expression in the fundus and lower segment were derived from non-myometrial cells. In contrast, the cellular composition of the cervix is more heterogeneous, and expression within the different cell types cannot be discerned. Nevertheless, we considered that maintenance of the physiological cellular environment was more important than a homogenous cell population.

Oxytocin and prostaglandins are known to have a fundamental role in human parturition [ 6]. Our gene array data are consistent with existing evidence on these oxytocics. Oxytocin is produced by the choriodecidua during human labor [ 8] and acts on myometrial oxytocin receptors to cause contraction. Since oxytocin is not produced in the myometrium but in other gestational tissues, it is reassuring that there was no increase in myometrial expression of oxytocin in our study. The increase in myometrial oxytocin receptor formation precedes the onset of labor, and uterine expression increases from mid-pregnancy to term rather than at the onset of labor [ 27]. Consistent with these data, we did not demonstrate an increase in oxytocin expression in labor. In contrast, we have previously demonstrated that expression of myometrial secretory phospholipase A2 is increased in samples taken after the onset of labor [ 15]. This enzyme catalyzes mobilization of arachidonic acid from membrane phospholipids for the synthesis of prostaglandins. Our gene array results confirm an increase in secretory phospholipase A2 expression in myometrial samples taken after the onset of labor and are consistent with a regulatory role for prostaglandins

There is no generally accepted statistical method to analyze differences in gene expressions between groups, due to correlation of expressions of individual genes. To validate our findings, we confirmed expression of genes with different technique and demonstrated functional relationship of co-expressed genes. We validated a proportion of our microarray findings by RT-PCR, and we were reassured that the results using both techniques were consistent among all tested genes. We also analyzed the patterns of expression by two techniques: K-means and hierarchical clustering. Although these techniques may not be completely independent, the method of gene clustering is different and hence the combination provides additional confidence for the identification of networks of co-regulated genes. Prior studies have shown that co-expressed genes have been demonstrated to be functionally related and to participate in common biological processes defined by the Gene Ontology database. These relationships are identified across species and functional categories [ 28– 31]. The identification within each cluster of genes with similar functions pertinent to labor strengthens our hypothesis that these genes are co-regulated. It is likely that expression of a particular gene can regulate expression of a second, which may itself influence a third. In this way, a single controlling mechanism may induce a multitude of phenotypic alterations leading to a change in function. Furthermore, transcription regulating factors (such as CAAT enhancer binding protein, CEBP, which is increased after labor in the lower segment) may promote transcription for numerous contraction-associated genes. Indeed, CEBP binding to the oxytocin receptor promoter has recently been demonstrated [ 32]. We anticipate that elucidating the networks of genes associated with labor will enable a more holistic understanding of the process, leading to more rational methods for manipulating uterine contraction.

In summary, we have demonstrated consistent changes in gene expression in the human lower segment, fundus, and cervix in association with labor. A number of novel-to-labor genes have been identified in addition to networks of co-expressed genes. There are marked tissue and spatial differences in gene expression in the uterus during parturition.

## Supporting Information

Dataset S1 Cervix: Selected Genes (565 KB XLS)

Dataset S2 Fundus: Selected Genes (558 KB XLS)

Dataset S3 Lower Segment: Selected (574 KB XLS)

### Accession Numbers

The GenBank ( http://www.ncbi.nlm.nih.gov/Genbank) accession numbers for the genes and gene products discussed in this paper are: alpha 1D–adrenergic receptor (M76446), beta-adrenergic receptor kinase 2 (AL022329), CEBP (M83667), calcitonin receptor activity–modifying protein 2 (AJ001015), calcium ion–transporting ATPase 2 (X63575), G protein–coupled receptor 161 (AI703188), G protein–coupled receptor 18 (L42324), GAPDH (U34995), guanine nucleotide-binding protein alpha (AC002077), interleukin-8 (M28130), oxytocin (NM_000915), phospholipase A2 IIA (M22430), prostaglandin receptor EP 4 (L28175), protein kinase C beta1(X07109), prothymosin alpha (M26708), REA (U72511), regulator of G protein signaling 2 (L13463), RXR (X52773), and tumor necrosis factor receptor (X60592).

## 

**Table 2:**
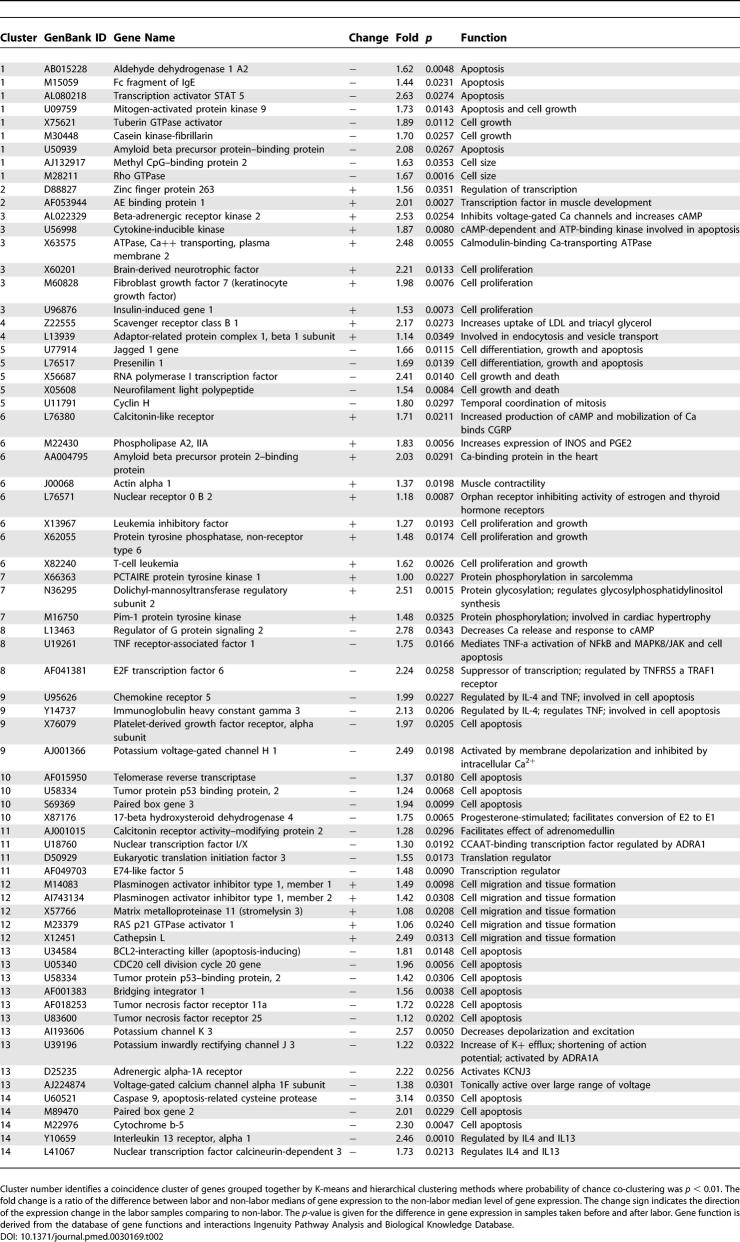
Lower Segment: Selected Genes within Each of the Coincidence Clusters with Functions Similar to Other Genes within the Same Cluster or Functions Pertinent to the Process of Labor

**Table 3:**
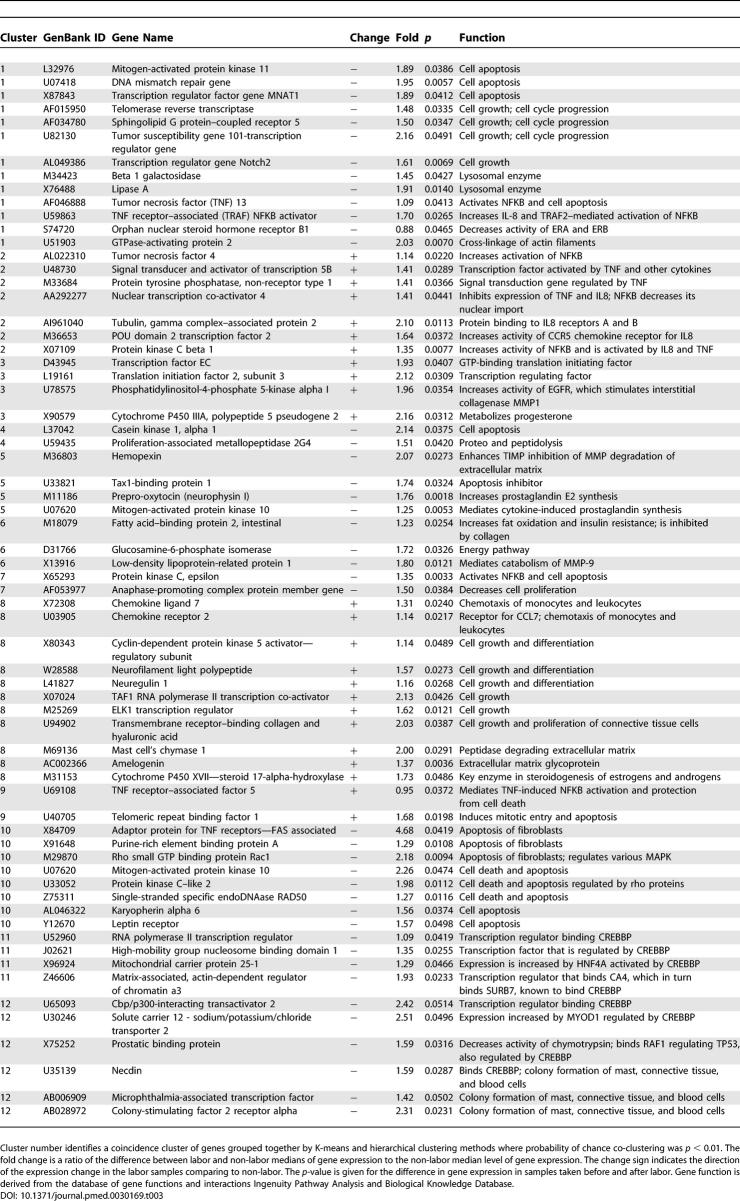
Cervix: Selected Genes within Each of the Coincidence Clusters with Functions Similar to Other Genes within the Same Cluster or Functions Pertinent to the Process of Labor

## Abbreviations

ERA: estrogen receptor alpha
GAPDH: glyceraldehyde-3-phosphate dehydrogenase
REA: repressor of estrogen receptor activity
RXR: retinoid X receptor alpha
